# *Akap1* Deficiency Promotes Mitochondrial Aberrations and Exacerbates Cardiac Injury Following Permanent Coronary Ligation via Enhanced Mitophagy and Apoptosis

**DOI:** 10.1371/journal.pone.0154076

**Published:** 2016-05-02

**Authors:** Gabriele Giacomo Schiattarella, Fabio Cattaneo, Gianluigi Pironti, Fabio Magliulo, Giuseppe Carotenuto, Marinella Pirozzi, Roman Polishchuk, Domenica Borzacchiello, Roberta Paolillo, Marco Oliveti, Nicola Boccella, Marisa Avvedimento, Maria Sepe, Assunta Lombardi, Rosa Anna Busiello, Bruno Trimarco, Giovanni Esposito, Antonio Feliciello, Cinzia Perrino

**Affiliations:** 1 Department of Advanced Biomedical Sciences, Federico II University, Naples, Italy; 2 Department of Internal Medicine (Cardiology), University of Texas Southwestern Medical Center, Dallas, Texas, United States of America; 3 Department of Physiology and Pharmacology, Karolinska Institute, Stockholm, Sweden; 4 Institute of Protein Biochemistry, Italian National Research Council (CNR-IBP), Naples, Italy; 5 Telethon Institute of Genetic and Medicine (TIGEM), Naples, Italy; 6 Department of Molecular Medicine and Medical Biotechnologies, Federico II University, Naples, Italy; 7 Department of Biology, Federico II University, Naples, Italy; Cleveland Clinic, UNITED STATES

## Abstract

A-kinase anchoring proteins (AKAPs) transmit signals cues from seven-transmembrane receptors to specific sub-cellular locations. Mitochondrial AKAPs encoded by the *Akap1* gene have been shown to modulate mitochondrial function and reactive oxygen species (ROS) production in the heart. Under conditions of hypoxia, mitochondrial AKAP121 undergoes proteolytic degradation mediated, at least in part, by the E3 ubiquitin ligase Seven In-Absentia Homolog 2 (Siah2). In the present study we hypothesized that *Akap1* might be crucial to preserve mitochondrial function and structure, and cardiac responses to myocardial ischemia. To test this, eight-week-old *Akap1* knockout mice (*Akap1*^-/-^), *Siah2* knockout mice (*Siah2*^*-/-*^) or their wild-type (*wt*) littermates underwent myocardial infarction (MI) by permanent left coronary artery ligation. Age and gender matched mice of either genotype underwent a left thoracotomy without coronary ligation and were used as controls (sham). Twenty-four hours after coronary ligation, *Akap1*^-/-^ mice displayed larger infarct size compared to *Siah2*^*-/-*^ or *wt* mice. One week after MI, cardiac function and survival were also significantly reduced in *Akap1*^-/-^ mice, while cardiac fibrosis was significantly increased. *Akap1* deletion was associated with remarkable mitochondrial structural abnormalities at electron microscopy, increased ROS production and reduced mitochondrial function after MI. These alterations were associated with enhanced cardiac mitophagy and apoptosis. Autophagy inhibition by 3-methyladenine significantly reduced apoptosis and ameliorated cardiac dysfunction following MI in *Akap1*^-/-^ mice. These results demonstrate that *Akap1* deficiency promotes cardiac mitochondrial aberrations and mitophagy, enhancing infarct size, pathological cardiac remodeling and mortality under ischemic conditions. Thus, mitochondrial AKAPs might represent important players in the development of post-ischemic cardiac remodeling and novel therapeutic targets.

## Introduction

Mitochondria are central to several cellular processes, as they are both the main source of energy and reactive oxygen species (ROS), and critical regulators of cell death or survival pathways [1]. Mitochondrial dysfunction has been observed in a variety of cardiac diseases, including coronary artery disease, left ventricular hypertrophy and heart failure [1–4]. During cardiac ischemia, mitochondrial ROS production represents one of the major determinants of infarct size and heart remodeling [4].

Recent studies have demonstrated the important role of a family of A Kinase Anchor Proteins (AKAPs) in the transmission of intracellular cyclic adenosine monophosphate (cAMP) signals to the outer membrane of mitochondria [3, 5]. Mitochondrial AKAPs (mitoAKAPs) are products of a single gene (*Akap1*), and are generated by alternative RNA splicing. All splice variants share the first 30 NH_2_-terminal residues mediating the targeting to the outer mitochondrial membrane and other functions, but diverge significantly at the C-terminus. Mitochondrial AKAP121 is transcriptionally regulated by cyclic adenosine monophosphate (cAMP) in vitro and in vivo [3]. Down-regulation of AKAP121 induced by cardiac pressure overload is associated with marked abnormalities in mitochondrial structure and function, and increased ROS generation [3]. In addition, AKAP121 undergoes a rapid post-translational degradation under hypoxic conditions mediated by the E3 ubiquitin ligase Seven In-Absentia Homolog 2 (*Siah2*) [6]. Siah2 also regulates the availability of hypoxia inducible factor-1α (HIF1-α) and other proteins [6–8], and its deletion prevents ischemia-induced cardiomyocyte cell death and reduces infarct size after coronary artery ligation [8]. These effects might be related, at least in part, to the regulation of AKAP121 availability and, in turn, of mitochondrial dynamics [8].

Stress-induced mitochondrial damage increases ROS production and can cause further damage to nearby mitochondria, the release of pro-apoptotic proteins and the amplification of cellular damage [9]. To prevent further injury, cells can induce the activation of quality control systems to remove damaged mitochondria by macro-autophagy or mitophagy [10, 11]. Selective degradation of damaged mitochondria may play an essential role in a number of cardiac pathologies associated with mitochondrial dysfunction [12–15]. Although it is now recognized the crucial role of autophagy in cardiovascular diseases, activation of autophagy mechanisms in cardiomyocytes might be beneficial or maladaptive depending on the timing and magnitude of the activation [16]. Whether mitoAKAPs might play a role in cardiac mitophagy is currently unknown. In the present study, we hypothesized that *Akap1* deletion might affect mitochondrial structure and function in the heart, and that mitoAKAPs levels might play a crucial role in the regulation of cardiomyocyte survival under conditions of ischemia and, in turn, cardiac remodeling and survival.

## Methods

### Experimental animals

All experiments involving animals were performed in conformity with the Guide for the Care and Use of Laboratory Animals published by the US National Institutes of Health (NIH Publication 8th edition, update 2011), and were approved by the animal welfare regulation of University Federico II, Naples, Italy. *Akap1*^-/-^ mice have been previously generated [17], and were donated by Stanley McKnight (University of Washington, Department of Pharmacology). *Siah2*^-/-^ mice have been previously generated, and were provided by kindly provided by David Bowtell PhD (Peter MacCallum Cancer Centre, Melbourne, Australia). Animals were maintained under identical conditions of temperature (21 ± 1°C), humidity (60 ± 5%) and light/dark cycle, and had free access to normal mouse chow.

### Transthoracic echocardiography

Cardiac function was non-invasively monitored by transthoracic echocardiography using the Vevo 770 high resolution imaging system using a 30-MHz RMV-707B scanning head (VisualSonics, Toronto, Canada) before the surgery and right before termination, one week after surgery, as previously described [18, 19].

### Mouse model of myocardial infarction

Mice were anesthetized with an intraperitoneal injection of 1 ml/kg (50 mg/kg) of a mixture of 50% tiletamine and 50% zolazepam (50 mg/ml tiletamine and 50 mg/ml zolazepam, Zoletil 100), plus xylazine 5 mg/Kg (Sigma-Aldrich) [18, 19]. The adequacy of anaesthesia was confirmed by the absence of reflex response to foot squeeze. Myocardial infarction (MI) was induced in *Akap1*^-/-^ (n = 27), *Siah2*^-/-^ (n = 27) and their wild type (*wt*) littermates (n = 32) by permanent coronary artery ligation as previously described [4]. Sham-operated animals underwent the same procedure without ligation of the left coronary artery. Two additional groups of *Akap1*^-/-^ or *wt* mice underwent systemic delivery by intraperitoneal injection of the autophagy inhibitor 3-methyladenine (3MA) one day before and every day after MI for one week (MI 1wk + 3MA, 40 mg/kg IP, n = 8 for both genotypes).

To determine infarct size, 24 hours after coronary artery ligation mice of both genotypes were anesthetized, the hearts were perfused with 1% Evans blue to determine the area at risk, and then removed. Each heart was sliced horizontally to yield four slices, and the slices were incubated in 1% triphenyltetrazolium chloride (TTC; Sigma-Aldrich) prepared with 200 mmol/L Tris buffer (pH 7.8) for 15 min at 37°C. With this procedure, viable non-ischemic myocardium stains blue, ischemic but still viable myocardium stains red, whereas the necrotic myocardium does not stain and appears pale white. The infarct area (IA, white), the area at risk (AAR, red and white) and the total left ventricle area (LV) were measured in each section using an image analyzer (Image J Software). IA/LV, AAR/LV and IA/AAR ratios were calculated and expressed as a percentage. In additional groups of mice, the hearts were harvested one hour, four hours and one week after coronary artery ligation and flash-frozen in liquid nitrogen to perform molecular analyses or fixed in specific fixatives to perform histological analyses (see below).

### Electron microscopy

Cardiac samples from animals of the different groups (n = 3/group) were fixed with 1% Glutaraldehyde (8% Aqueous Solution, Electron Microscopy Sciences) in Sodium Cacodylate buffer 0.2 M pH 7.4. The specimens were washed in distilled water, stained with 2% Osmium tetroxide and 0.5% Uranyl acetate, dehydrated through a graded series of ethanol ethanol (50, 70, 90, and 100%), Propylene Oxide and embedded in Epon resin (Sigma) and polymerized at 60°C for 72 h. Ultrathin (60 nm) sections were cut with a Leica EM UC7 ultramicrotome (Leica Microsystems GmbH, Wetzlar, Germany) and mounted on grids. Images were acquired from thin sections using a FEI Tecnai 12 transmission electron microscope (FEI Company, Hillsboro, Oregon, USA) equipped with a Veleta CCD digital camera (Olympus Soft Imaging Solutions GmbH, Münster, Germany). Count and quantification of mitochondria was performed using the iTEM software (Soft Imaging Systems GmbH, Münster, Germany). For quantification, 15 random regions for each sample were considered.

### Cardiac histology

Mouse heart specimens were fixed in 4% formaldehyde and embedded in paraffin. After de-paraffinization and re-hydratation, 4 μm-thick sections were prepared, mounted on glass slides and stained with Sirius Red as previously described [4]. To quantify myocardial capillary density, lectin staining was used to specifically stain endothelial cells, as previously described [20].

### TUNEL staining

The DNA nicks were determined with the use of an in situ Apoptosis Detection kit or ApopTag Fluorescein Direct in Situ Apoptosis Detection kit (Chemicon) according to manufacturer’s instructions as previously described [4, 21]. TUNEL staining was visualized by specific green fluorescence and nuclei by 4'-6-diamidino-2-phenylindole (DAPI). The number of TUNEL-positive cardiomyocytes nuclei was counted, and data were normalized per total nuclei identified by DAPI staining in the same sections (n = 7–8 animals/group). Equal numbers of cells were analyzed per group, and statistical analysis was performed on myocyte groups by 2-way ANOVA.

### Protein extraction and Western Blotting

Left ventricular samples were homogenized in a buffer containing 50 mM Tris–HCl (pH 7.4), 150 mM NaCl, 1% Nonidet P40, 1 mM EDTA, 0.25% sodium deoxycholate, 10 mM NaF, 10 μM Na_3_VO_4_, 1 mM phenylmethylsulfonylfuoride, and a protease inhibitor cocktail (10 g/ml aprotinin, 10 g/ml pepstatin, 10 g/ml leupeptin) [22] using the program Protein_1 on a GentleMACS tissue Dissociator (Miltenyi Biotec). Lysates were centrifuged at 14,000 rpm for 15 min and protein concentrations in all lysates were measured using a dye-binding protein assay kit (Bio-Rad) and a spectrophotometer reader (Biorad) at a wavelength of 595 nm. Immunoblotting was performed using commercially available antibodies anti-PARP-1 (mouse monoclonal, Santa Cruz), p62 (rabbit polyclonal, Cell Signaling) and α-Tubulin (mouse monoclonal, Santa Cruz). Secondary antibodies were purchased from Amersham Life Sciences Inc. Bands were visualized by enhanced chemiluminescence (ECL; Amersham Life Sciences Inc.) according to the manufacturer’s instructions, and were quantified using densitometry (Chemidoc, Biorad, USA). All experiments and densitometric quantifications were separately repeated at least three times.

### Mitochondrial ROS generation and aconitase activity

Mitosox Red (Molecular Probes) was used to assess the generation of mitochondrial superoxide in vivo as previously described [4]. Changes in mitochondrial aconitase activity were also evaluated as previously described [4].

### Mitochondria isolation and detection of respiratory parameters

Mitochondrial respiration rate was detected on isolated mitochondria from *Akap1*^-/-^ (n = 4), *Akap1*^+/-^ (n = 4) or *wt* (n = 4) hearts as previously described [4]. Heart tissue fragments were gently homogenized in 10 vol of an isolation medium consisting of 220 mM mannitol, 70 mM sucrose, 20 mM Tris·HCl, 1 mM EDTA, and 5 mM EGTA (pH 7.4). Then, 0.1 mg protease/g tissue (nagarse 12 U/mg protein) was added to the homogenate and incubated for 4 min at 4°C, The homogenate was then centrifuged at 8,000 g for 10 min at 4°C. The pellet was resuspended in 10 vol of isolation medium (supplemented with 0.5% fatty acid free BSA), subjected to very gentle homogenization, and centrifuged 500 g for 10 min at 4°C. The resulting supernatant was centrifuged at 8,000 g, in order to obtain the mitochondrial pellet, that was washed twice, re-suspended in a minimal volume, and kept on ice until further determinations.

Mitochondrial respiration was measured polarographically by using a Clark type oxygen electrode (Rank Brothers) at 37°C in 0.5 mL of respiratory buffer (KCl 80 mmol/L, HEPES 50 mmol/L, EGTA 1 mmol/L, KH_2_PO_4_ 5 mmol/L, MgCl_2_ 2 mmol/L, 0.5% BSA, pH 7.0). After recording basal respiration, state 2 respiration was initiated by adding glutamate 5 mmol/L and malate 2.5 mmol/L as substrates for complex I or succinate 5 mmol/L (in combination with rotenone 2 μmol/L to inhibit complex I) as substrate for complex II. Complex IV respiration was determined after the addition of antimycin A (1.8 μmol/L) to the respiratory buffer in order to inhibit complex II and TMPD (*N*,*N*,*N'*,*N'*-tetramethyl-*p*-phenylenediamine) 300 μmol/L and ascorbate 3 mmol/L, which donates electrons to cytochrome oxidase via the reduction of cytochrome c. State 3 respiration was determined after the addition of 300 μmol/L ADP.

### Statistical analysis

All data presented are expressed as mean ± SE and are representative of three or more independent experiments. Comparisons between 2 groups were performed using the unpaired Student *t* test. For MI experiments, comparisons were made by 2-way analysis of variance (ANOVA) or, when noted, by 1-way ANOVA, and *p* values shown indicate the effect of genotype on the MI-stimulated response. Correction for multiple comparisons was made using the Student–Newman–Keuls method. A minimum value of *p*<0.05 was considered statistically significant. Kaplan-Meier survival analysis was performed using GraphPad Prism version 6.01 (GraphPad Software, Inc. La Jolla, CA), and differences were considered to be statistically significant at a value of P <0.05 by log-rank test.

## Results

### *Akap1* deletion enhances infarct size and exaggerates adverse cardiac remodeling and mortality after permanent coronary artery ligation

To investigate the functional role of mitoAKAPs in the heart, we studied mice with global genetic deletion of the *Akap1* gene (*Akap1*^-/-^) and their *wt* littermates. *Akap1*^-/-^ mice appear normal and healthy, even if their body weight is lower compared to their *wt* littermates (Table 1). Under basal conditions or after the sham operation, cardiac morphometry and function of *Akap1*^-/-^ or *Akap1*^+/-^ mice was not significantly different from *wt* mice (Table 1). To test whether *Akap1* deletion might influence mitochondrial respiratory pathways, we determined respiration rates by using three different substrates, such as piruvate+malate, succinate+rotenone, TmPD+ ascorbate + antimycine in *Akap1*^-/-^, *Akap1*^+/-^ and *wt* mice. These substrates allowed us to evaluate the activity of complex I, complex II and complex IV-linked respiratory pathways, respectively. Mitochondrial respiration rates were not statistically different between the groups (S1 Fig), whatever the substrate considered, thus indicating that *Akap1* deletion, partial or total, does not influence cardiac mitochondrial functionality under basal conditions.

**Table 1:** Cardiac morphometry and echocardiography of mice from the different groups.

	sham				MI 1wk		
	*wt*	*Siah2*^*-/-*^	*Akap1*^*+/-*^	*Akap1*^*-/-*^	*wt*	*Siah2*^*-/-*^	*Akap1*^*-/-*^
	*(n = 8)*	*(n = 9)*	*(n = 7)*	*(n = 8)*	*(n = 8)*	*(n = 10)*	*(n = 7)*
BW, g	27.1 ± 0.9	26.3 ± 1.0	24.3 ± 1.0	23.4 ± 1.3^#^	27.4 ± 1.5	26.8 ± 1.4	24.6 ± 3.3
LVW, mg	85.6 ± 2.7	83.4 ± 3.2	76.8 ± 2.5	75.9 ± 3.8	90.7 ± 8.1*	95.8 ± 6.2*	95.6 ± 7.4*
LAW, mg	4.8 ± 0.3	5.1 ± 0.5	5.9 ± 0.5	6.3 ± 0.7	7.1 ± 2.3	8.1 ± 1.6	13.3 ± 2.6*^§^
HW, mg	106.4 ± 2.5	103.7 ± 2.8	96.0 ± 2.5	98.3 ± 5.1	117.1 ± 6.4*	105.8 ± 7.8	131.5 ± 10.7*^§^
LVW/BW	3.1 ± 0.1	3.2 ± 0.2	3.2 ± 0.1	3.0 ± 0.1	3.3 ± 0.2	3.6 ± 0.2	3.9 ± 0.2*^§^
HW/BW	3.9 ± 0.1	3.8 ± 0.1	4.0 ± 0.1	4.1 ± 0.1	4.3 ± 0.3	4.3 ± 0.2	5.3 ± 0.2*^§^
LVEDd, mm	3.1 ± 0.1	3.1 ± 0.1	3.0 ± 0.1	3.0 ± 0.1	3.6 ± 0.2*	3.4 ± 0.1	3.7 ± 0.3*
LVESd, mm	1.3 ± 0.1	1.3 ± 0.1	1.2 ± 0.1	1.2 ± 0.1	2.2 ± 0.2*	1.9 ± 0.1*	2.7 ± 0.3*^§^
IVSd, mm	0.8 ± 0.1	0.7 ± 0.1	0.8 ± 0.1	0.8 ± 0.1	0.6 ± 0.1	0.7 ± 0.1	0.6 ± 0.1
PWd, mm	0.8 ± 0.1	0.8 ± 0.1	0.8 ± 0.8	0.8 ± 0.1	0.7 ± 0.1	0.8 ± 0.1	0.7 ± 0.1
FS, %	57.7 ± 2.0	58.1 ± 1.8	60.7 ± 2.3	56.7 ± 1.8	39.1 ± 4.4*	45.1 ± 3.1*	28.3 ± 3.3*^§^
HR, bpm	462 ± 26	484 ± 20	546 ± 35	498 ± 10	556 ± 47	508 ± 22	513 ± 33

**Abbreviations used**: Body weight, **BW**; left ventricle weight, **LVW**, left atrium weight, **LAW**; heart weight, **HW**; left ventricular end-diastolic diameter, **LVEDd**; left ventricular end-systolic diameter, **LVESd**; interventricular septum end-diastolic diameter, **IVSd**; posterior wall end-diastolic diameter, **PWd**; fractional shortening, **FS**; heart rate, **HR** (^#^p<0.05 vs. correspondent *wt*; *p<0.05 vs. *wt* sham; ^§^p<0.05 vs. *wt* MI).

**Fig 1:**
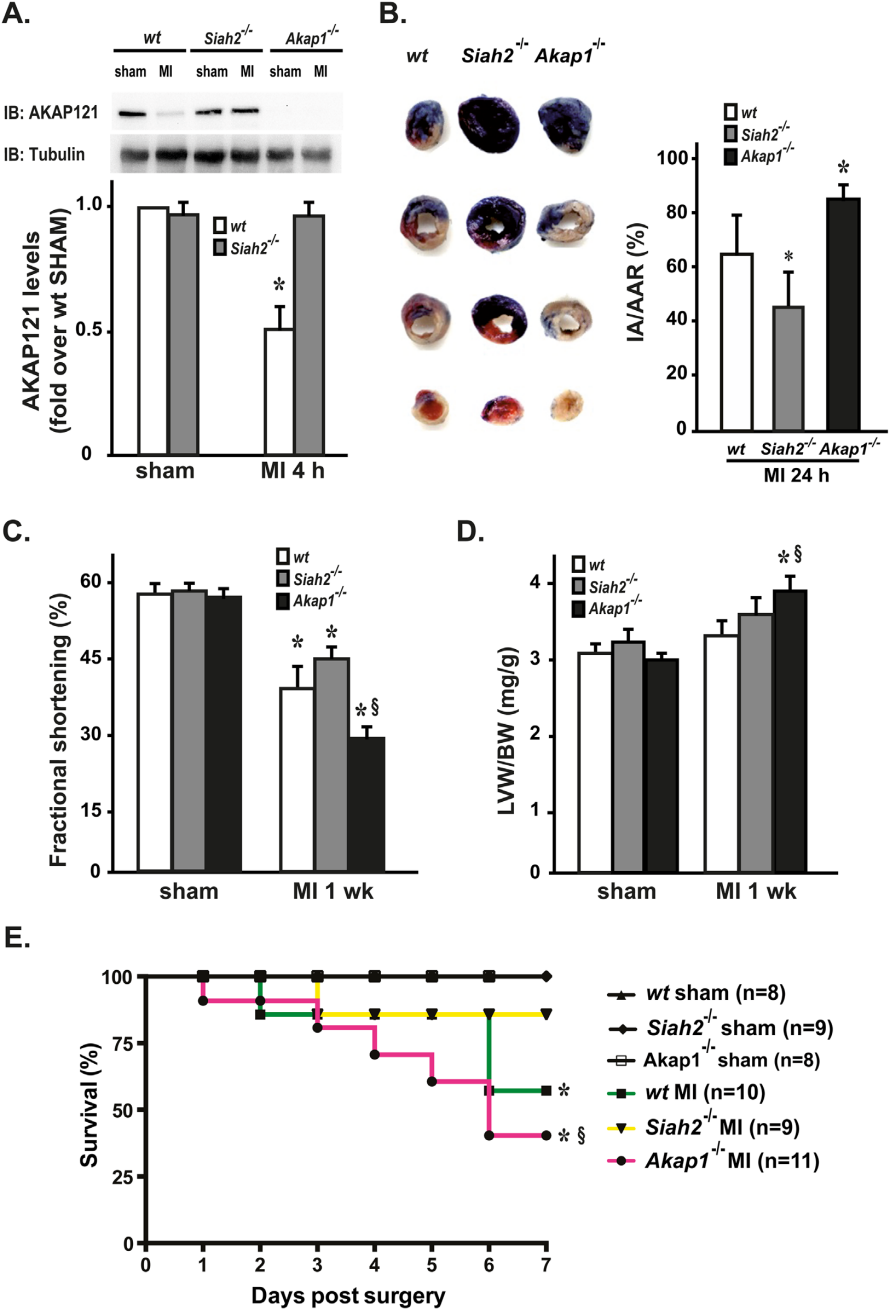
*Akap1* genetic deletion increases infarct size and reduces survival after myocardial infarction. **(A)** Representative immunoblot (*top*) and densitometric analysis (*bottom*) of 4 independent experiments to evaluate AKAP121 protein levels in heart samples from wild type (*wt*), *Siah2*^*-/-*^ and *Akap1*^-/-^ mice after the sham procedure or after 4 hours of permanent coronary artery ligation (MI 4 h) (*p<0.05 vs. sham; n = 5 hearts/group). Tubulin protein levels did not significantly change among the samples. **(B) *Left*:** Representative images of TTC staining of heart sections from *wt*, *Akap1*^-/-^ or *Siah2*^*-/-*^ mice 24 hours after MI. ***Right*:** Bar graphs showing ratios of myocardial infarct area (IA) over area at risk (AAR) in MI mice from all the groups (*p<0.05 vs. *wt* MI). **(C)** Cumulative data of % fractional shortening 1 week after the sham or MI procedures (MI 1 wk) in *wt*, *Akap1*^-/-^ or *Siah2*^*-/-*^ mice (*p<0.05 vs. sham; ^§^p<0.05 vs. *wt* MI). **(D)** Bar graphs showing cumulative data of left ventricular weight (LVW) to body weight (BW) ratio 1 week after the sham or MI procedures in *wt*, *Akap1*^-/-^ or *Siah2*^*-/-*^ mice (*p<0.05 vs. sham; §p<0.05 vs. wt MI). **(E)** Kaplan–Meier cumulative survival analysis of *wt*, *Akap1*^-/-^ or *Siah2*^*-/-*^ mice after the sham (*wt*: n = 8; *Siah2*^*-/-*^: n = 9; *Akap1*^-/-^: n = 8) or MI procedures (*wt*: n = 10; *Siah2*^*-/-*^: n = 9; *Akap1*^-/-^: n = 11, *p<0.05 vs. sham; §p<0.05 vs. *wt* MI).

To test the role of mitoAKAPs under conditions of myocardial ischemia, myocardial infarction (MI) was induced by permanent coronary artery ligation in *wt*, *Akap1*^-/-^ and *Siah2*^*-/-*^ mice. Sham-operated animals from all genotypes underwent the same surgical procedure without occlusion of the coronary artery (Table 1). Four hours after MI, *wt* mice displayed a significant reduction in cardiac AKAP121 levels (Fig 1A) that persisted up to four weeks after MI (data not shown). As expected, AKAP121 down-regulation was significantly inhibited in *Siah2*^*-/-*^ mice (Fig 1A). Twenty-four hours following permanent coronary artery ligation, hearts were infused with Evans blue to demarcate the ischemic area susceptible to infarction (area at risk, AAR), and counterstained with triphenyltetrazolium chloride (TTC) to identify the final infarct area (IA) from the viable myocardium within the AAR. Compared to *wt*, *Akap1*^-/-^ hearts demonstrated a significantly larger proportion of infarcted myocardium within the LV and the AAR, and the AAR displayed a not statistically significant increasing trend (Table 2). In contrast, consistent with previous results [8], the IA/AAR ratio was significantly lower in *Siah2*^*-/-*^ mice (Fig 1B and Table 2).

**Table 2:** Infarct area and area at risk 24 hours after permanent coronary artery ligation in mice from the different experimental groups.

	*wt (n = 12)*	*Siah2*^-/-^ *(n = 9)*	*Akap1*^-/-^ *(n = 4)*
**IA/LV (%)**	21.8 ± 7.7 (s.e. = 2.2)	16.6 ± 3.4 (s.e. = 1.1)	42.6 ± 9.1* (s.e. = 4.5)
**IA/AAR (%)**	64.2 ± 15.4 (s.e. = 4.4)	45.1 ± 13.5^#^ (s.e. = 4.5)	84.9 ± 4.4* (s.e. = 2.2)
**AAR/LV (%)**	32.8 ± 14.1 (s.e. = 4.1)	38.3 ± 8.4 (s.e. = 2.8)	49.5 ± 9.0 (s.e. = 4.5)

**Abbreviations used:** infarct area, **IA**; area at risk, **AAR**; left ventricle area, **LV**. Data are mean ± standard deviation; standard error (s.e.) is also reported for each group (*p<0.05 vs. *wt*; ^#^*p*<0.05 vs. *wt* and *Akap1*^*-/-*^).

Since infarct size is one of the major determinants of adverse post-ischemic remodeling, cardiac function was evaluated by trans-thoracic echocardiography in *wt*, *Akap1*^-/-^ and *Siah2*^*-/-*^ mice one week after MI. In contrast to *Siah2*^*-/-*^ mice, *Akap1*^-/-^ mice displayed a significant worsening in cardiac function after coronary artery ligation (Fig 1C), and a more pronounced increase in left ventricular weight (LVW) to body weight (BW) ratio at study termination compared to *wt* MI mice (Fig 1D and Table 1).

To assess whether these differences would translate into a survival benefit, survival rates were monitored for seven days in all the groups after the surgery. As expected, survival of sham mice was 100%, while MI resulted in a lower survival in all genotypes compared to the respective sham group. Interestingly, the 7-day-survival of *Akap1*^-/-^ MI mice was significantly lower compared to *wt* or *Siah2*^*-/-*^ MI mice (Fig 1E).

### Increased apoptosis and fibrosis in *Akap1*^*-/-*^ hearts after myocardial infarction

**Fig 2:**
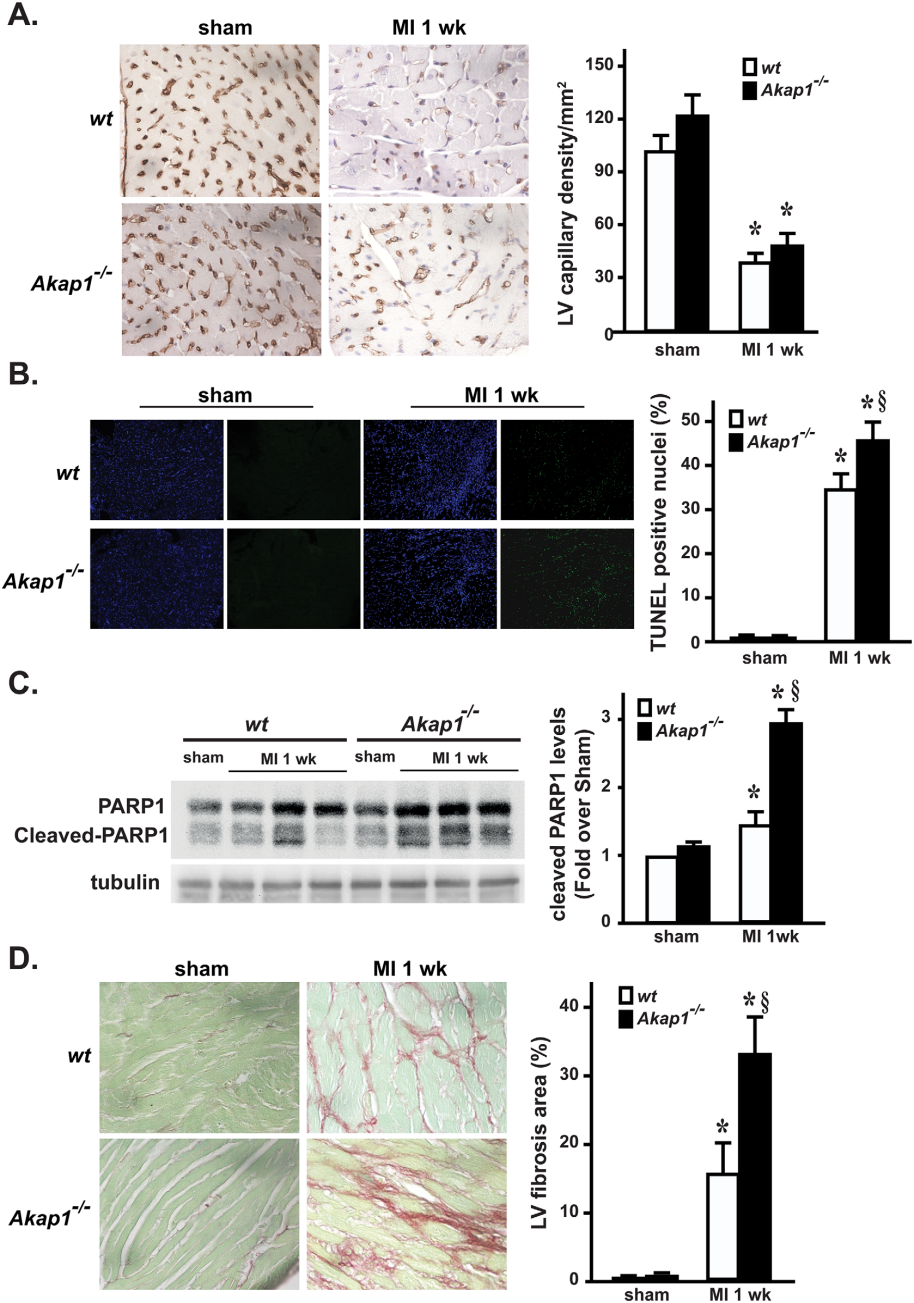
*Akap1* genetic deletion enhances apoptosis and fibrosis after myocardial infarction. **(A) *Left*:** Representative lectin staining of cardiac sections from *wt* or *Akap1*^-/-^ hearts after the sham procedure or 7 days of permanent coronary artery ligation (MI 1wk). Capillaries appear brown. ***Right*:** Bar graphs of cumulative data of multiple independent experiments analyzing capillary density in the different groups (*p<0.05 vs. sham; n = 4 hearts/groups). **(B) *Left*:** Representative DAPI and TUNEL staining of cardiac sections from *wt* or *Akap1*^-/-^ mice after the sham procedure or MI 1wk. Positive nuclei appear green. ***Right*:** Bar graphs of cumulative data of multiple independent experiments on TUNEL staining (*p<0.05 vs. sham; §p<0.05 vs. *wt* MI; n = 4 hearts/group). **(C)** Representative immunoblots (l*eft*) and densitometric analysis (*right*) of 4 independent experiments to evaluate cleaved PARP-1 protein levels in *wt* and *Akap1*^-/-^ mice after the sham or MI 1wk procedures. Tubulin protein levels did not significantly change among the samples (*p<0.05 vs. sham; §p<0.05 vs. wt MI; n = 5 hearts/group). **(D) *Left*:** Representative images of Sirius red staining of cardiac sections from *wt* or *Akap1*^-/-^ mice 7 days after the sham procedure or MI (60X magnification). ***Right*:** Bar graphs showing cumulative data of multiple independent experiments analyzing percent fibrosis in peri-infarct areas (*p<0.05 vs. sham; §p<0.05 vs. *wt* MI; n = 4 hearts/groups).

To determine whether abnormal vascularization might be responsible for the increased infarct size in *Akap1*^-/-^ mice, histological studies were performed in the peri-infarct zone to stain cardiac capillaries in all experimental groups. Although coronary anatomy was not determined, sham *Akap1*^-/-^ mice displayed a cardiac capillary density similar to *wt* (Fig 2A). As expected, coronary artery ligation induced a significant reduction in myocardial capillary density in both *Akap1*^-/-^ and *wt* mice, and no significant differences among MI groups were observed (Fig 2A).

To assess whether enhanced apoptotic cell death might be involved in the increased infarct size observed in *Akap1*^-/-^ mice, terminal deoxynucleotidyl transferase dUTP nick end labeling (TUNEL) staining was performed in cardiac sections from the different groups. Compared to MI, sham hearts from both groups displayed a significant increase in the rate of TUNEL positive nuclei in the infarct zone (Fig 2B). However, MI *Akap1*^-/-^ mice displayed a significantly increased rate of apoptotic nuclei compared to MI *wt* (Fig 2B). Consistent with these observations, cardiac levels of cleaved Poly (ADP-ribose) polymerase (PARP), a marker of apoptosis, were significantly higher in MI *Akap1*^-/-^ mice compared to MI *wt* (Fig 2C). The increased rates of apoptotic cell death observed after MI in *Akap1*^-/-^ mice were also associated to a significant increase in interstitial fibrosis after MI compared to *wt* (Fig 2D). Consistent with previous in vitro observations [23] *Akap1*^-/-^ MI hearts were also characterized by enhanced cardiomyocyte hypertrophy in the peri-infarct zone, as shown by increased cross-sectional area (S2 Fig).

### *Akap1* deletion induces mitochondrial aberrations and enhances cardiac mitophagy

**Fig 3:**
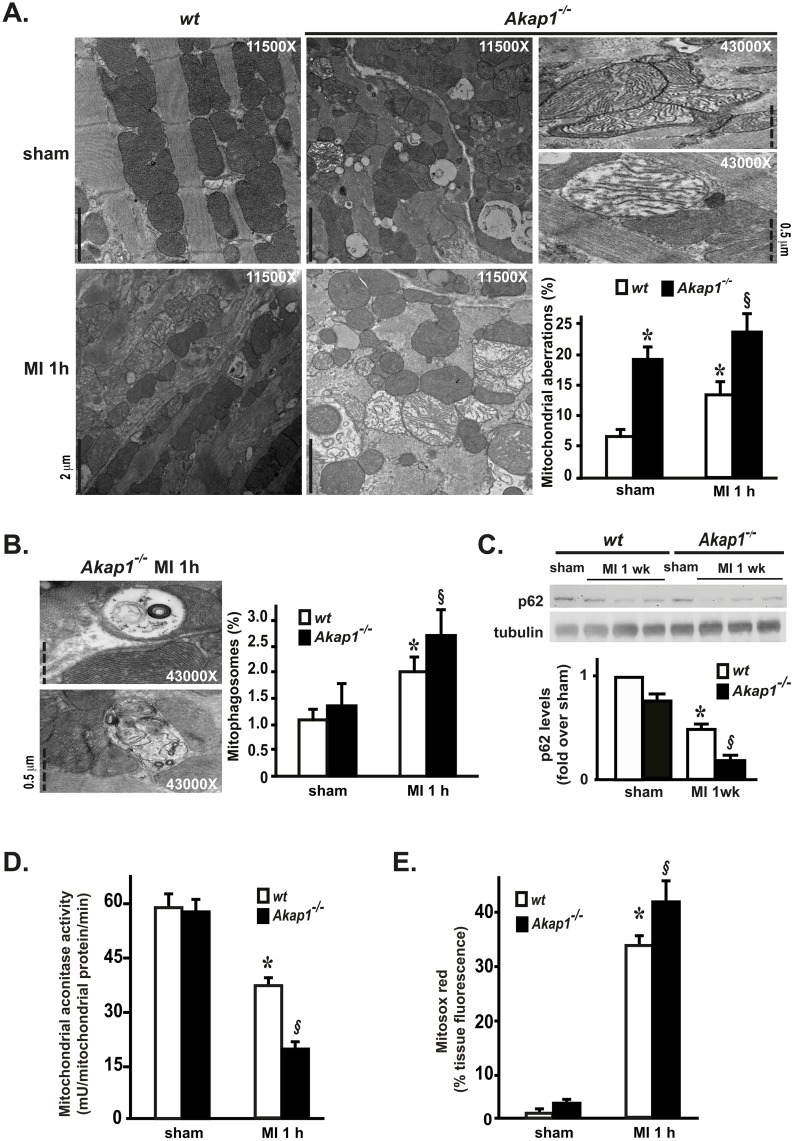
*Akap1* genetic deletion promotes mitochondrial dysfunction and enhances mitophagy after myocardial infarction. **(A)** Representative electron microscopy images of mitochondria in cardiac sections from *wt* or *Akap1*^-/-^ mice after the sham procedure or one hour of permanent coronary artery ligation (MI 1h) and bar graphs showing cumulative data of multiple independent experiments to quantify % mitochondrial aberrations (n = 3 hearts/group; scale bars and magnifications are reported in the single panels; *p<0.05 vs. *wt* sham; §p<0.05 vs. wt MI). **(B) *Left*:** Representative electron microscopy images of mitophagosomes observed in *Akap1*^-/-^ MI 1h mice. Magnifications and scale bars are reported in the single panels. ***Right*:** Bar graphs showing quantitative data on % mitophagosomes observed in *wt* or *Akap1*^-/-^ after the sham or MI 1h procedures (*p<0.05 vs. sham; §p<0.05 vs. wt MI). **(C)** Representative immunoblot and densitometric analysis of 4 independent experiments to evaluate p62 protein levels in *wt* and *Akap1*^-/-^ mice after the sham or MI 1wk procedures. Tubulin protein levels did not significantly change among the samples (*p<0.05 vs. sham; §p<0.05 vs. wt MI; n = 6 hearts/group). **(D)** Bar graphs showing cumulative data of multiple independent experiments measuring mitochondrial aconitase activity in *wt* or *Akap1*^-/-^ cardiac mitochondria after the sham or MI 1h procedures (*p<0.05 vs. sham; §p<0.05 vs. wt MI; n = 4 hearts/group). **(E)** Bar graphs showing cumulative data of multiple independent experiments to assess mitochondrial ROS generation in *wt* or *Akap1*^-/-^ heart sections after the sham or MI 1h procedures (*p<0.05 vs. sham; §p<0.05 vs. *wt* MI 1h; n = 4 hearts/group).

We have previously shown that cardiac AKAP121 downregulation under conditions of pressure overload is associated with mitochondrial abnormalities [3]. To determine whether *Akap1* deletion might directly affect mitochondrial structure, we next investigated mitochondrial morphology at electron microscopy in *Akap1*^-/-^ mice and their *wt* littermates, either after the sham operation or one hour MI. In sham *wt* hearts, mitochondria displayed normal size and shape, and were organized in rows perpendicular to the myocardial Z-lines as expected (Fig 3A, top left panel). In contrast, mitochondria in *Akap1*^-/-^ sham hearts were more disorganized around the fibers, and were characterized by significant alterations in the architecture, including mitochondrial swelling, matrix dilution, reduced number and/or fragmentation of mitochondrial cristae (Fig 3A, top middle panel). One hour after coronary artery ligation, a significant increase in the percent number of aberrant mitochondria was observed in *wt* MI hearts (Fig 3A, bottom left panel), and these alterations were significantly higher in *Akap1*^-/-^ MI hearts (Fig 3A, bottom middle and right panels). After one hour MI, in both genotypes there was an increase in aberrant mitochondria sequestered within double-membrane structures (mitophagosomes), and in MI *Akap1*^-/-^ hearts the percent number of mitophagosomes was significantly higher than MI *wt* (Fig 3B). Consistent with these results, endogenous p62 levels, a ubiquitin-binding scaffold protein that co-localizes with ubiquitinated protein aggregates and is usually degraded during autophagy or mitophagy [24], were significantly reduced in *wt* MI hearts after one week compared to sham, and further reduced in *Akap1*^-/-^ MI hearts compared to *wt* MI (Fig 3C).

Consistent with these results, despite the severe morphological mitochondrial abnormalities in sham *Akap1*^-/-^ hearts, aconitase activity was not statistically different compared to *wt* (Fig 3D). However, aconitase activity was significantly reduced after MI in both genotypes, and was significantly lower in *Akap1*^-/-^ MI hearts compared to *wt* (Fig 3D). Similarly, mitochondrial ROS production, measured by Mitosox staining, was not statistically different among the sham groups, while it increased after MI in both genotypes, and was significantly higher in *Akap1*^-/-^ MI hearts compared to *wt* (Fig 3E).

### Inhibition of autophagy reduces apoptosis and ameliorates cardiac dysfunction in *Akap1*^*-/-*^ mice

**Fig 4:**
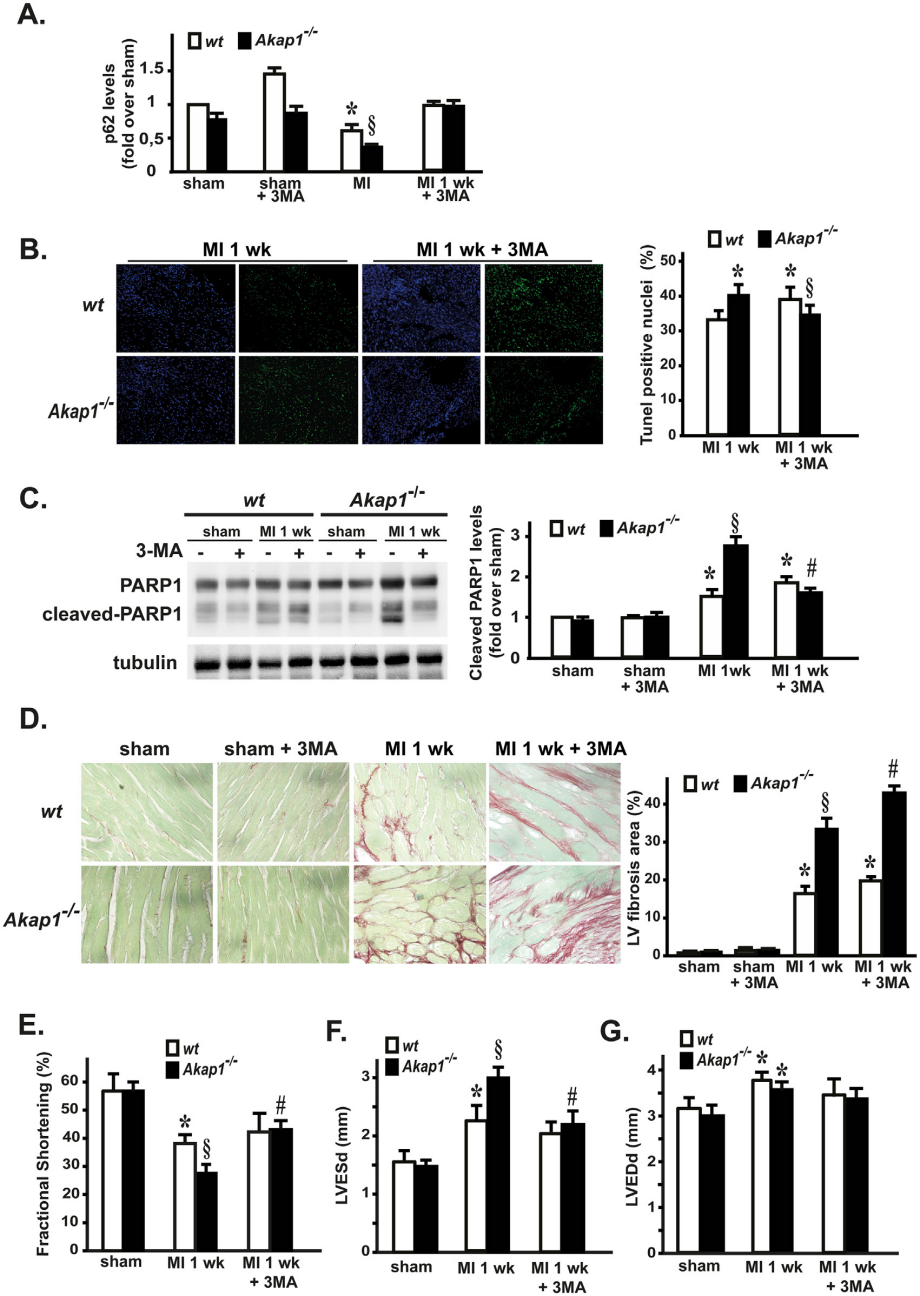
Inhibition of autophagy reduces apoptosis and ameliorates cardiac dysfunction in *Akap1*^-/-^ mice. **(A)** Densitometric analysis of 3 independent experiments to evaluate p62 protein levels in *wt* and *Akap1*^-/-^ mice after the sham procedure or 1 week of permanent coronary artery ligation (MI 1 wk), either untreated (-) or treated (+) with 3-methyladenine (3MA). Tubulin protein levels did not significantly change among the samples (*p<0.05 vs. sham; §p<0.05 vs. *wt* MI; n = 5 hearts/group). **(B) *Left*:** Representative DAPI and TUNEL staining of cardiac sections from *wt* and *Akap1*^-/-^ MI 1wk mice, untreated or treated with 3MA (20X magnification). Positive nuclei appear green. ***Right*:** Bar graphs representation of cumulative data of multiple independent experiments (*p<0.05 vs. *wt* MI; §p<0.05 vs. *wt* MI 1wk ± 3MA; n = 4 hearts/group). **(C)** Representative immunoblot (*left*) and densitometric analysis (*right*) of three independent experiments to evaluate cleaved PARP-1 protein levels in *wt* or *Akap1*^-/-^ mice after the sham or MI 1wk procedure, ±3MA. Tubulin protein levels did not significantly change among the samples (*§p<0.05 vs. sham; §p<0.05 vs. *wt* MI 1wk; ^#^p<0.05 vs. *Akap1*^-/-^ MI 1wk; n = 5 hearts/group). **(D) *Left*:** Representative images of Sirius red staining of cardiac sections from *wt* and *Akap1*^-/-^ mice after MI (1wk), untreated or treated with 3MA (60X magnification). ***Right*:** Bar graphs summarizing cumulative data of multiple independent experiments (*p<0.05 vs. sham; §p<0.05 vs. *wt* MI 1wk; ^#^p<0.05 vs. *Akap1*^-/-^ MI 1wk; n = 4 hearts/group). **(E)** Cumulative data of % fractional shortening **(E)**, left ventricular end-systolic dimensions (LVESD) **(F)** and left ventricular end-diastolic dimensions (LVEDD) **(G)** in *wt* or *Akap1*^-/-^ mice undergoing the sham, MI 1wk or MI 1wk + 3MA procedures (for all, *p<0.05 vs. sham; §p<0.05 vs. *wt* MI 1wk; ^#^p<0.05 vs. *Akap1*^-/-^ MI 1wk).

In order to determine whether autophagy inhibition might exert beneficial effects in *Akap1*^-/-^ hearts and prevent at least some of the detrimental features induced by MI, *wt* and *Akap1*^-^ mice were pre-treated with the autophagy inhibitor 3-methyladenine (3MA) for three days, and subsequently underwent the sham or MI one week procedures. As shown in Fig 4A, 3MA efficiently prevented p62 degradation in both *wt* and *Akap1*^-/-^ MI mice. Interestingly, 3MA treatment reduced apoptotic cell death in *Akap1*^-/-^ mice after MI (Fig 4B and 4C), and although it increased myocardial fibrosis in the same group (Fig 4D), it significantly improved cardiac dysfunction in *Akap1*^-/-^ mice one week after MI, as shown by a significant increase in % fractional shortening (Fig 4E). This beneficial effect was mainly due to a reduction in left ventricular end-systolic dimensions (LVESd) (Fig 4F), while left ventricular end-diastolic dimensions (LVEDd) were unchanged in 3MA-treated MI mice compared to untreated animals (Fig 4E).

## Discussion

This paper demonstrates, for the first time, that *Akap1* is required to preserve mitochondrial structure in the heart, since its genetic deletion results in mitochondrial morphological abnormalities and enhanced mitophagy. Under conditions of myocardial ischemia, *Akap1* deletion increases cardiomyocyte mitophagy and apoptosis, enhances infarct size and adverse cardiac remodeling, ultimately reducing survival after myocardial infarction. Autophagy inhibition reduces apoptosis and ameliorates cardiac dysfunction in a *Akap1*^-/-^ mice, suggesting that mitochondrial damage and enhanced mitophagy in this model might be responsible, at least in part, for the development of the pathological phenotype.

Targeting of cAMP-dependent protein kinase (PKA) by mitoAKAPs plays a major role in mitochondrial pathophysiology, generating local signal transduction units that include signaling enzymes, adaptor molecules and mRNAs [7]. Under myocardial ischemia, AKAP121 levels were reduced most likely as consequence of enhanced degradation by the hypoxia-induced E3 ligase Siah2 [6–8]. Indeed, genetic deletion of *Siah2* prevented AKAP121 degradation after coronary artery ligation, and this effect was associated with reduced infarct size and enhanced survival after MI in *Siah2*^*-/-*^ mice compared to *wt*. These results suggest that modulation of AKAP121 levels by *Siah2* during myocardial ischemia might be crucial for cardiomyocyte survival and, in turn, for post-ischemic cardiac remodeling and survival.

By using a genetic model of *Akap1* global deletion, we directly investigated the role of mitoAKAPs in the regulation of mitochondrial structure and function in the heart under basal conditions or after myocardial infarction [8]. *Akap1*^-/-^ or *Akap1*^+/-^ mice appear normal, and even if *Akap1*^-/-^ mice presented lower body weight compared to their *wt* littermates, under resting conditions their heart rate, LV/BW and HW/BW ratios were not significantly different from the different genotypes. Moreover, cardiomyocyte cross-sectional area in *Akap1*^-/-^ sham hearts was similar to sham *wt* (S2 Fig), and cardiac mitochondrial respiration was normal under basal conditions in both *Akap1*^-/-^ or *Akap1*^+/-^ mice (S1 Fig). Despite this apparently normal phenotype, *Akap1*^-/-^ cardiomyocytes displayed remarkable mitochondrial morphological aberrations, without evidence of increased apoptosis or fibrosis under basal conditions.

After permanent coronary artery ligation, *Akap1*^-/-^ MI mice displayed enhanced mitophagy, ROS production and apoptosis compared to *wt* MI mice. Cardiac fibrosis and cardiomyocyte hypertrophy in the peri-infarct zone was also significantly increased in these mice compared to MI *wt*. Although studies in isolated adult cardiomyocyets were not performed, it is possible to speculate that basal mitochondrial disarray observed in *Akap1*^-/-^ hearts, albeit compensated under basal conditions, increases cardiomyocyte susceptibility to ischemia, enhancing cardiomyocyte death, accelerating cardiac dysfunction and in turn reducing post-MI survival.

Although the role of cardiomyocytes autophagy during cardiovascular disease is well documented [25–28], less is known about mitochondrial autophagy (mitophagy) in the regulation of cardiomyocytes survival and remodeling [2, 29, 30]. Our findings unveil the function of *Akap1* in the control of mitophagy under ischemic conditions, and suggest that mitoAKAPs might represent another important example of mitochondrial proteins crucially involved in cardiac adaptations to pathological stimuli [2]. In the absence of *Akap1*, mitochondrial abnormalities likely increased cardiac mitophagy and, by enhancing ROS production, enhanced cardiomyocytes apoptosis during ischemia. Interestingly, autophagy inhibition did not exert beneficial effects in *wt* mice undergoing MI, and rather increased apoptosis while not affecting cardiac fibrosis in this group of mice. Taken together, these results suggest that a delicate (and complex) balance regulating mitochondrial dynamics might be required to preserve cardiac function during hypoxia, and while inhibition of “exaggerated” mitophagy in *Akap1*^-/-^ hearts is beneficial, the same molecular intervention, under different conditions, might be neutral or even detrimental.

Although mitochondrial alterations of morphology and function observed in *Akap1*^-/-^ hearts are likely attributable to the reduced targeting of PKA and associated complexes on the outer mitochondrial membrane, the precise molecular signaling pathways involved in the development of mitochondrial structural abnormalities and mitophagy are currently not known. Several molecular partners have been previously described for mitoAKAPs, as well as important mitochondrial PKA substrates [31, 32]. The presence of the N-terminus extra-sequence that switches localization of AKAP149 from mitochondria to the endoplasmic reticulum [33–35] suggests that targeting the scaffold complex on distinct organelles might also represent a strategic and rapid mechanism to efficiently redirect cAMP signaling to distinct intracellular routes. Importantly, we cannot exclude that some of the features observed in *Akap1*^-/-^ mice might be dependent, at least in part, on the differential subcellular localization of the splicing variants to the sarcoplasmic reticulum also. The extra-mitochondrial effects of *Akap1* global deletion must be taken into account and deserve future investigation.

In conclusion, our findings highlight the critical role of mitochondrial AKAPs in cardiac responses to ischemia, modulating mitochondrial structure, ROS production and mitophagy and ultimately infarct size, cardiac remodeling and survival after MI. Therefore, *Akap1* might represents a novel important therapeutic target in ischemic cardiac diseases.

## Supporting Information

S1 Fig Mitochondrial respiratory parameters in hearts from *wt*, *Akap1*^*-/-*^ and *Akap1*^*+/-*^ mice. Mitochondrial respiratory parameters [State 2, State 3, and respiratory control ratio (RCR)] in hearts from *wt*, *Akap1*^-/-^ and *Akap1*^*+/-*^ mice. **(A)** Parameters were detected in presence of Complex I-linked substrate (Piruvate+malate), Complex II-linked substrate (succinate+rotenone) **(B)**, Complex IV linked substrate (TmPD + ascorbate + antimycin) **(C)**. Values represent mean ± SE of 3–4 different hearts, each one performed in duplicate.
(TIF)

S2 Fig Cardiomyocyte cross-sectional area in cardiac sections from *wt* or *Akap1*^*-/-*^ mice after the sham procedure or 1 week MI. **(A)** Representative microphotographs of cardiac cross-sections from wt and *Akap1*^*-/-*^ mice undergoing the sham procedure (sham) or 1-week myocardial infarction (MI 1 wk) stained with wheat germ agglutinin (WGA) and counterstained with DAPI. Scale bar = 100 μm. **(B)** Quantification of cardiomyocytes area (*p<0.05 vs. sham wt; §p<0.05 vs. all; n = 3–4 animals/group).
(TIF)
