# Correction: *Akap1* Deficiency Promotes Mitochondrial Aberrations and Exacerbates Cardiac Injury Following Permanent Coronary Ligation via Enhanced Mitophagy and Apoptosis

**DOI:** 10.1371/journal.pone.0158934

**Published:** 2016-07-06

**Authors:** Gabriele Giacomo Schiattarella, Fabio Cattaneo, Gianluigi Pironti, Fabio Magliulo, Giuseppe Carotenuto, Marinella Pirozzi, Roman Polishchuk, Domenica Borzacchiello, Roberta Paolillo, Marco Oliveti, Nicola Boccella, Marisa Avvedimento, Maria Sepe, Giuseppe Gargiulo, Assunta Lombardi, Rosa Anna Busiello, Bruno Trimarco, Giovanni Esposito, Antonio Feliciello, Cinzia Perrino

Dr. Giuseppe Gargiulo is not included in the author byline. He should be listed as the fourteenth author and affiliated with Federico II University, Naples, Italy. The contributions of this author are as follows: Performed the experiments.

## References

[1] Schiattarella GG, Cattaneo F, Pironti G, Magliulo F, Carotenuto G, Pirozzi M, et al (2016) *Akap1* Deficiency Promotes Mitochondrial Aberrations and Exacerbates Cardiac Injury Following Permanent Coronary Ligation via Enhanced Mitophagy and Apoptosis. PLoS ONE 11(5): e0154076 doi:10.1371/journal.pone.0154076 27136357 10.1371/journal.pone.0154076 PMC4852950

