# Supplementary figures and images for: Akap1 Deficiency Promotes Mitochondrial Aberrations and Exacerbates Cardiac Injury Following Permanent Coronary Ligation via Enhanced Mitophagy and Apoptosis
Source: PLoS One. 2016 May 2;11(5):e0154076. doi: 10.1371/journal.pone.0154076 (PMC4852950; doi:10.1371/journal.pone.0154076)

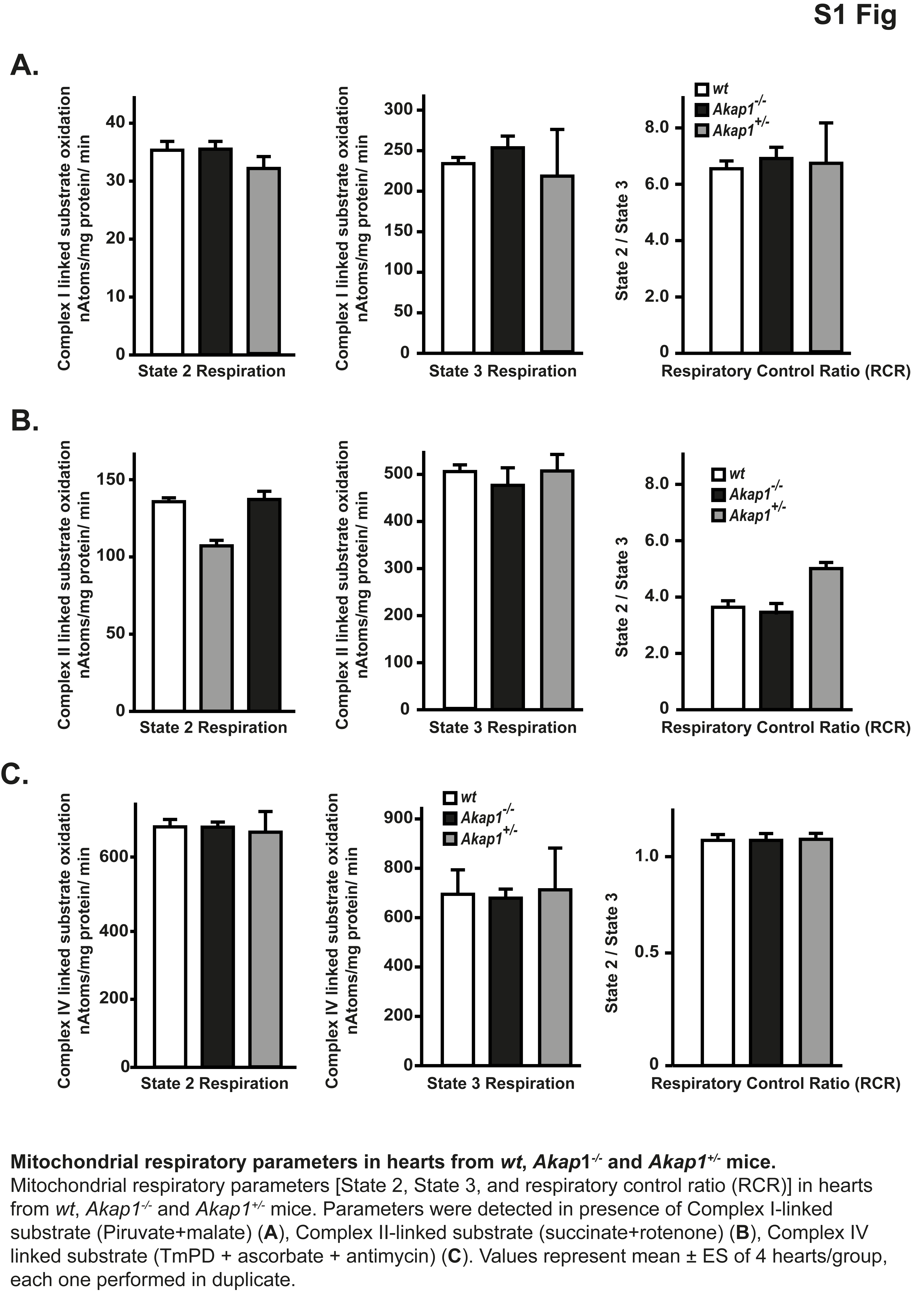

Supplement: S1 Fig — Mitochondrial respiratory parameters [State 2, State 3, and respiratory control ratio (RCR)] in hearts from wt, Akap1-/- and Akap1+/- mice. (A) Parameters were detected in presence of Complex I-linked substrate (Piruvate+malate), Complex II-linked substrate (succinate+rotenone) (B), Complex IV linked substrate (TmPD + ascorbate + antimycin) (C). Values represent mean ± SE of 3–4 different hearts, each one performed in duplicate. (TIF) [file pone.0154076.s001.tif]

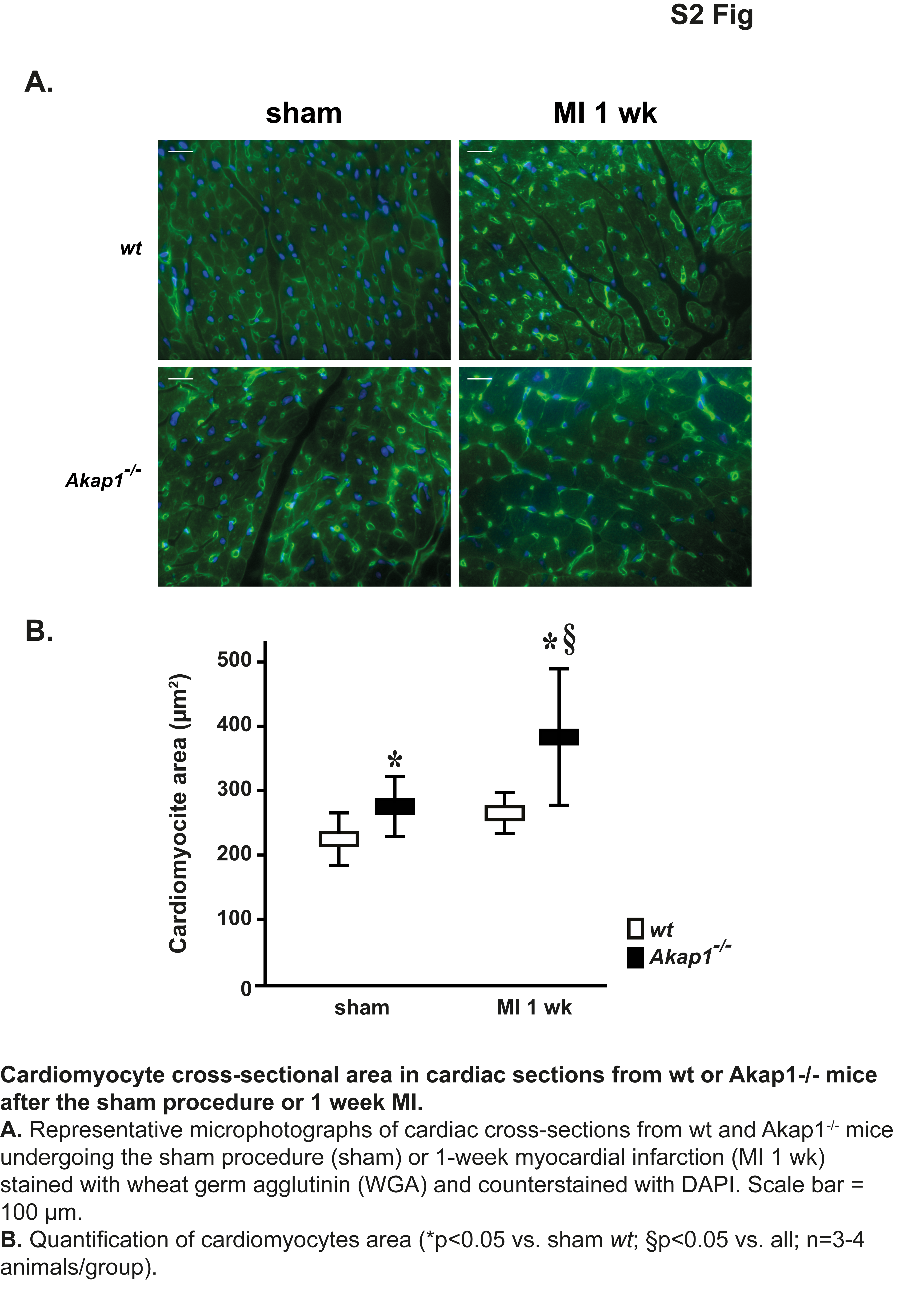

Supplement: S2 Fig — (A) Representative microphotographs of cardiac cross-sections from wt and Akap1-/- mice undergoing the sham procedure (sham) or 1-week myocardial infarction (MI 1 wk) stained with wheat germ agglutinin (WGA) and counterstained with DAPI. Scale bar = 100 μm. (B) Quantification of cardiomyocytes area (*p<0.05 vs. sham wt; §p<0.05 vs. all; n = 3–4 animals/group). (TIF) [file pone.0154076.s002.tif]
